# Distinctness of Brazilian common bean cultivars with carioca and black grain by means of morphoagronomic and molecular descriptors

**DOI:** 10.1371/journal.pone.0188798

**Published:** 2017-11-30

**Authors:** Jessica Delfini, Vânia Moda-Cirino, Claudete de Fátima Ruas, José dos Santos Neto, Paulo Maurício Ruas, Juliana Sawada Buratto, Eduardo Augusto Ruas, Leandro Simões Azeredo Gonçalves

**Affiliations:** 1 Agronomy Department, Universidade Estadual de Londrina (UEL), Rodovia Celso Garcia Cid, Londrina, Brazil; 2 Instituto Agronômico do Paraná (IAPAR), Rodovia Celso Garcia Cid, Londrina, Brazil; 3 Biology Department, Universidade Estadual de Londrina (UEL), Rodovia Celso Garcia Cid, Londrina, Brazil; Università Politecnica delle Marche, ITALY

## Abstract

Common bean (*Phaseolus vulgaris* L.) is one of the most important legumes for human consumption and is a staple food in the diet of the population of some countries of Latin America, Africa and Asia. The distinction between cultivars is based predominantly on morphological descriptors, which proved inefficient for the differentiation of some cultivars. This study had the objective of describing, distinguishing and evaluating the agronomic potential of 39 common bean cultivars of the carioca and black grain groups registered for cultivation in Brazil, based on 49 morphoagronomic descriptors and microsatellite (simple sequence repeat -SSR) markers. The morphoagronomic traits of each commercial group were characterized in four environments. Thirty-seven SSR markers were used for the molecular description. The morphological data, analyzed by the Shanonon-Weaver index, detected low variability among cultivars for qualitative data. On the other hand, the estimates of variance analysis, relative importance of the traits and hierarchical grouping analysis applied to the quantitative variables showed that the descriptors related to plant morphology were the most important for the carioca group, and those related to seed morphology were determining for the black group. The genetic parameters estimated for SSR markers by hierarchical and Bayesian cluster analysis identified 116 alleles, with 33 and 30 polymorphic loci and 24 and 22 private alleles for the carioca and black groups, respectively. The combined use of morphoagronomic and molecular descriptors improves the distinguishability of cultivars, contributing in a more efficient way to breeding and to the protection of cultivars.

## Introduction

Common bean (*Phaseolus vulgaris* L.) is a staple in the diet of the population of some countries of Latin America, Africa and Asia, and a notable source of proteins, carbohydrates, fibers, and minerals [1–3]. Worldwide, around 12 million tons of common beans are produced per year, and Brazil is one of the main producers [4]. In the harvest of 2015/2016, the Brazilian production was 3.33 million tons with a mean yield of 1.103 Kg ha^-1^ [5].

The nationwide level of common bean productivity in Brazil was significantly optimized in the last decades, mainly due to new production technologies and the development of new, increasingly productive cultivars, with stable yields and well-adapted to the diversified production systems. According to data of the Ministry of Agriculture, Livestock and Food Supply (MAPA), 330 common bean cultivars are registered for cultivation, mainly developed by public companies, e.g., the Brazilian Agricultural Research Corporation (EMBRAPA), Instituto Agronômico do Paraná (IAPAR) and Instituto Agronômico de Campinas (IAC) [6].

In Brazil, the registration of cultivars is controlled by Seed Law no. 10.711/03. One of the requirements for the registration of a cultivar is that the Value for Cultivation and Use (VCU), determined and confirmed in experiments, fulfills minimal species-specific criteria. On the other hand, the national crop protection service (SNPC) is in charge of protecting intellectual property, ensuring exclusivity in the rights of commercial exploitation and use of royalties. The SNPC has a proper legislation, related to international intellectual property laws, controlled by the International Union of Protection of Plant Varieties (UPOV) [7].

The most important requirements for cultivar protection include the tests of Distinguishability, Homogeneity and Stability (DHS), which check whether the candidate cultivar satisfies the technical requirements, according to the criteria of SNPC. Currently, the requirement for the distinction between cultivars is based on morphological descriptors, which proved efficient for cultivar differentiation, except in some cases of species with a narrow genetic base.

Molecular markers are considered important tools for the identification of cultivars, for allowing polymorphism analysis directly in the DNA [8,9]. In this respect, microsatellite or SSR markers have being extremely useful for cultivar distinction, particularly in the case of species with a narrow genetic base, where DNA fingerprinting may be highly efficient. The application of SSR marker in genetic studies of the genus *Phaseolus* is paved, since numerous primers were developed and optimized for use in the literature [10–14].

Molecular descriptors may help to determine the varietal purity and commercial control of the seeds of protected cultivars, and may be complementary to the morphological descriptors used in the DHE assays. Thus, the objective of this study was to describe, distinguish and evaluate the agronomic potential of 39 common bean cultivars registered in Brazil, employing morphoagronomic descriptors and microsatellite markers.

## Materials and methods

### Plant material

For the morphoagronomic and molecular characterization, 39 common bean cultivars were used, 20 of which belong to the commercial group carioca (IAPAR 81, IPR Eldorado, IPR Tangará, IPR Campos Gerais, IPR Curió, IPR Andorinha, IPR Maracanã, IPR Bem-te-vi, IPR Quero-quero, Pérola, BRS Estilo, BRS Notável, Carioca, IAC Alvorada, IAC Formoso, IAC Imperador, FT 65, TAA Bola Cheia, TAA Gol, and TAA Dama), and 19 to the black group (IAPAR 8 –Rio Negro, IAPAR 20, IAPAR 44, IAPAR 65, Rio Tibagi, IPR Uirapuru, IPR Chopim, IPR Graúna, IPR Gralha, IPR Tuiuiú, IPR Nhambu, BRS Valente, BRS Campeiro, BRS Supremo, BRS Esteio, IAC Una, IAC Diplomata, FT Soberano, and FT 41).

These cultivars were chosen because they are widely sown by the farmers in the main cultivation areas in Brazil. They were developed in breeding programs of public or private institutions and registered by the National Register of Cultivars of the Ministry of Agriculture, Livestock and Supply—RNC/MAPA (S1 and S2 Tables). The cultivars were characterized separately for each commercial group.

### Morphoagronomic characterization

The study included a total of 49 descriptors, proposed by the International Union for the Protection of New Varieties of Plants (UPOV) and selected based on the official descriptors of common bean (*Phaseolus vulgaris* L.) established by the Brazilian Ministry of Agriculture, Livestock and Supply MAPA and published in the Diário Oficial da União—DOU on November 5, 1997.

The following qualitative morphological descriptors were used: presence of anthocyanin in cotyledons, hypocotyl and stem; plant type; color of the central leaflet of the 4^th^ plant node; leaf roughness; color of the flower, wing, banner, and seed; position of the terminal inflorescence; color uniformity of the pod; primary and secondary pod color; pod profile; pod apex; apical tooth shape; apical tooth position on the pod; venation of the seed coat; seed brightness; presence of seed halo; seed halo color; and commercial seed group (http://www.upov.int). The quantitative descriptors included: primary leaf length (PLL); primary leaf width (PLW); primary leaf index (PPL/PLW) (PLI); central leaflet length (CLL); central leaflet width (CLW); central leaflet index (CLL/CLW) (CLI); main stem length (StL); insertion height of the 1^st^ pod (IFP); number of nodes on the main stem (NN); pod length (PL); number of seeds per pod (SP); number of locules per pod (LP); total number of pods per plant (NPP); total number of seeds per plant (NSP); main stem thickness (StTh); seed length (SL); seed width (SWth); seed thickness (STh); total seed weight per plant (TSW); weight of 1,000 seeds (W1000); coefficient J (COEF J); coefficient H (COEF H); and grain yield (YLD).

The experiments of morphoagronomic characterization were installed in four environments; two in the rainy season of 2014/2015, with sowing between September and October, in Ponta Grossa (25^o^09’11”S; 50^o^09’22”W; altitude: 869 m asl) and in Guarapuava (25^o^23’51”S; 51^o^32’36”W; altitude: 1041 m asl), and two in the dry season of 2015, sown between January and February, in Ponta Grossa and Santa Tereza do Oeste (25^o^05’20”S; 53^o^35’25”W, altitude: 750 m asl). The experiments were arranged in a randomized complete block design with three replications and plots consisting of four 4-m rows spaced 0.5 m apart, at a density of 12 plants per linear meter, considering the two central rows for evaluation.

### Molecular characterization

A 25-seed sample of each of the 39 cultivars was sown in 5 pots in a greenhouse of the Experimental Station of IAPAR, Londrina, Paraná, to obtain 8–10 plants per cultivar. When the plants reached growth stage V4, the second trifoliolate leaf of each of the cultivars was collected and stored at -80 ^ο^C. The DNA was extracted according to the protocol of [15]. DNA concentration of was estimated using the NanoDrop 2000/2000c spectrophotometer (Thermo Scientific, USA) and samples were diluted to a final concentration of 10 ng/μl.

Each cultivar was analyzed based on a bulk mixture, containing samples of 8–10 plants with the same DNA concentration. When only one allele was observed in the bulk, the cultivar was considered pure. However, when more than one allele was identified at at least two loci, the cultivar was considered heterogeneous, and the bulk was opened, i.e., each one of the plants of the bulk was genotyped individually for the locus marker in heterozygosity. This procedure was used to verify if there was heterozygosity at the locus or homozygosity for different alleles in different plants, e.g., cases of mixtures of pure lines [16].

The genotyping system was developed from a group of 37 microsatellite primers pairs developed specifically for common bean [10–14] (S3 Table). These primers were selected because they represent loci with a high polymorphism content, good genomic distribution and specific amplification pattern.

Microsatellite loci were amplified according to a strategy proposed by [17]. To this end, an M13 tail, consisting of 18 nucleotides (3’ TGTAAAACGACGGCCAGT 5’) was added to the 5 'end of the forward primers. This same sequence was synthesized, labeled with one of the three fluorophores (6-FAM, HEX and NED). For the multiplex system, groups of up to three primers with different amplification sizes were formed to avoid interference between them, and at the time of the PCR reaction, each primer was labeled with a different fluorophore. All PCR reactions were carried out in a final volume of 10 μl containing: 4.5 μl of Green Taq mix (Green Master mix 1X, Promega, USA); 0.08 μl forward primer (concentration 5 pM); 0.32 μl reverse primer (concentration 5 pM); 0.32 μl of the M13 primer labeled with a different fluorophore (FAM, HEX or NED); 2 μl of DNA (10 ng/μl); and 2.78 μl ultra pure water.

The amplification was performed in the PTC200 thermocycler (MJ Research, USA) in a program consisting of an initial denaturation stage at 94°C for 4 min, followed by 10 touchdown cycles, in which the annealing temperature decreased 1°C per cycle. Each cycle consisted of three stages: denaturation (30 sec at 94°C), annealing (30 sec starting at 65°C and ending with 55°C at the 10^th^ cycle) and extension (30 sec at 72°C). Another 30 cycles were added to this program, consisting of: denaturation (30 sec at 94°C), annealing (30 sec at 55°C) and extension (30 sec at 72°C), followed by seven cycles consisting of denaturation (30 sec at 94°C), annealing (45 sec at 53°C) and extension (45 sec at 72°C); plus a final extension step at 60°C for 40 min.

Amplification products, obtained with each of three distinct primers pairs and labeled with the fluorophores FAM, NED or HEX, were pooled (3 μL of each reaction) in a single mixture. Then, 1 μl of this mixture was combined with 0.2 μL of the molecular weight standard GeneScan ™ 600 LIZ Size Standard (Life Technologies, USA) and adjusted to 10 μL with 8.8 μL HI-DI formamide (Life Technologies, USA), for multiplexed capillary electrophoresis, using in the automated 3500xL DNA Genetic Analyzer (Applied Biosystems, USA). The fluorescence peaks were visualized with the GeneMarker program, version 2.4.0 (Soft Genetics) for genotyping.

### Statistical analysis

For the morphoagronomic traits, frequency distribution analyses and variability were estimated using the Shannon-Weaver index (H'index) [18]. For the quantitative traits, individual variance analysis was performed for each environment, followed by the analysis of combined variance considering all environments, preceded by the test of homogeneity of variances by the Hartley method. In the combined analysis of variance, the effects of genotypes and environments were considered fixed. Correlations between the variables were estimated by Pearson’s correlation analysis.

The quantitative morphoagronomic data were subjected to multivariate analyses, considering the mean of the cultivars in the four environments. The generalized distance of Mahalanobis was used and then clusters were grouped by Ward’s method. The relative importance of the quantitative variables studied was analyzed by the [19]. The analysis of variance, Hartley method, relative importance, and genetic distances were processed using software Genes [20], the grouping and Pearson's correlation was performed by the R program (http://www.r-project.org), using the packages *dendextend* [21] and *corrplot* [22], respectively.

The molecular data were analyzed using software Popgene [23] to estimate the number of alleles per locus (*A*), number of effective alleles (*Ae*), number of polymorphic loci, expected (*H*_*E*_) and observed heterozygosity (*H*_*O*_), Shannon index (*I*) and Nei’s genetic distance [24], and then subjected to cluster analysis by Ward’s method, using the R program (http://www.r-project.org). The correlations between morphoagronomic and molecular matrices were performed by R program, using the package *dendextend*.

Software Cervus version 2.0 [25] was used to estimate the polymorphic information content (*PIC*), and the possibility of null alleles (*An*). The number of private alleles was determined using Genetic Data Analysis (GDA) [26]. The linkage disequilibrium, the Analyses of Molecular Variance (AMOVA), and the fixation index (Fst) were performed using Arlequin v.3.1.1 [27]. The genetic structure analysis was performed by Bayesian methods with software STRUCTURE v.2.2 [28]. Delta K [29] was used to determine the number of genetically homogeneous groups with software Structure Harvester [30]. A Bayesian method was conducted with BayesAss 3.0 to estimate the gene flow probability among the cultivars within and between groups, by the distributions of immigration rates [31].

## Results and discussion

### Morphoagronomic characterization

Analysis of qualitative morphoagronomic descriptors, as proposed by UPOV, revealed low variability between cultivars of the carioca and black grain groups, with a mean H'index of 0.14 and 0.19, respectively. No anthocyanin was detected in the cotyledons, hypocotyl and stem of any cultivar of the carioca group, while the black-grain cultivars tested positive for anthocyanin. The two groups showed uniform flower color, white wing and banner in the case of the carioca cultivars, and purple for the black cultivars. The pod color at physiological maturity of some cultivars of the black group was uniform, while in the carioca group the color was uniform for all cultivars. Both groups had an irregular pod color at harvest maturity, pods with abrupt apex, marginal position of the apical tooth, and no seed halo. The carioca cultivars had an uneven seed color and presence of venation on the seed coat, while the cultivars of the black group had regularly colored seeds and no venation on the seed coat.

The breeding programs aim to meet the demands of the consumer market, seeking new cultivars with agronomic and culinary traits more accepted in each region-specific, using only parents that satisfy this standard in the crosses. Thus, the low variability among the cultivars may be related to the narrowing of the genetic base of these cultivars, considering that the methods used in Brazilian breeding programs are conservative and exploit mainly Mesoamerican and little exotic germplasm. These methods consist of the hybridization of improved superior lines and selection by the genealogical, population or single-seed descent method. Currently, some breeding programs have used recurrent selection to broaden the genetic base of the cultivars and increase selection gains [32].

The individual analysis of variance of the quantitative data showed significance for most analyzed variables, indicating the existence of genetic variability among the carioca and black cultivars. The homogeneity test of variances indicated that the residual variances for almost all studied variables were homogeneous. Only the variable YLD in the carioca and black groups, and SWth and STh in the black group showed that variance was not homogeneous, and adjustments of the degrees of freedom for an adequate performance of the combined analysis of variance were necessary [33].

The combined analysis of variance of the black and carioca groups identified a significant effect of genotypes (G) for all variables except YLD in the black group (Tables 1 and 2). For the effect of environments (E) only the variables NN and LP were not significant for the carioca and black groups, respectively, whereas for the GA interaction, no significance was detected for the variables YLD and StTh of the carioca group, or for the variables NSP, SL, SWth, STh, and YLD of the black group. These results demonstrate the differentiated behavior of the genotypes against the alterations of the environments for most of the analyzed variables.

**Table 1:** Combined variance analysis for 23 agromorphological traits evaluated in the characterization study of common bean cultivars of the commercial group carioca in four environments in the state of Paraná in the rainy season of 2014/15 and dry season of 2015.

FV^1/^	Mean Square								Mean	CV(%)	>MSr/ <MSr
	Block/	Cultivars (C)		Environment (E)		CxE		Error			
	Environment										
PLL	1.174	1.503	**	113.521	**	0.298	**	0.118	6.7	5.13	1.8
PLW	1.216	1.164	**	21.012	**	0.167	**	0.084	5.39	5.38	3.17
PLI	0.003	0.015	**	2.752	**	0.003	**	0.001	1.24	3.56	5.57
CLL	0.891	1.721	**	59.195	**	0.468	**	0.294	9.19	5.9	2.98
CLW	0.78	1.351	**	39.778	**	0.284	**	0.183	7.14	6	3.29
CLI	0.004	0.042	**	0.028	*	0.003	**	0.002	1.29	3.54	2.38
StL	70.569	3053.3	**	12657.79	**	179.574	**	77.739	79.46	11.1	5.25
IFP	4.34	22.132	**	373.76	**	8.662	**	4.691	15.45	14.02	1.46
NN	1.811	17.688	**	4.894	ns	2.425	**	1.399	13.89	8.52	2.46
PL	0.065	3.426	**	8.227	**	0.195	**	0.117	11.27	3.04	2.37
NSP	0.112	1.425	**	3.396	**	0.255	**	0.151	5.96	6.53	1.51
LP	0.083	1.357	**	1.1	**	0.135	**	0.077	6.62	4.2	2.08
NPP	17.804	41.562	**	1379.066	**	26.385	**	12.661	20.15	17.66	5.86
NSP	454.106	1806.5	**	19864.49	**	564.818	**	267.168	90.18	18.12	5.88
StTh	0.166	2.836	**	16.929	**	0.232	ns	0.236	6.17	7.88	1.58
SL	0.103	1.073	**	6.607	**	0.101	**	0.049	10.76	2.07	2.4
SWth	0.058	0.449	**	2.291	**	0.034	**	0.016	6.82	1.89	3.03
STh	0.045	0.381	**	3.869	**	0.073	**	0.023	5.11	3.02	2.29
TSW	34.503	131.289	**	1404.112	**	47.991	**	19.254	23.5	18.67	4.19
W1000	136.155	4177.36	**	47693.87	**	647.246	**	223.185	261.69	5.71	2.84
COEF J	0.0006	0.027	**	0.007	**	0.001	**	0.0006	1.58	1.67	3
COEF H	0.0003	0.005	**	0.032	**	0.001	**	0.00045	0.75	2.84	2
YLD^(2)^	58778.47	147926	**	1858847	**	61367.52	ns^(42)^	44619^(99)^	2275.7	23.21	10.53

*/** significant at 5% and 1% probability, respectively

ns = not significant, Test F, DF Blocks/Environment = 8, DF Cultivars = 19, DF Environment = 3, DF CxE = 57, DF Residue = 152, ^1/^PLL: primary leaf length (cm); PLW: primary leaf width (cm); PLI: primary leaf index (PLL/PLW); CLL: central leaflet length (cm); CLW: central leaflet width (cm); CLI: central leaf index (CLL/ CLW); StL: main stem length (cm); IFP: insertion height of the first pod (cm), NN: number of stem nodes, PL: pod length (cm); NSP: number of seeds per pod; LP: number of locules per pod; NPP: number of pods per plant; NSP: number of seeds per plant; StTh: main stem thickness (mm); SL: seed length; SWth: Seed width (mm); STh: seed thickness; TSW: total seed weight in the plant (g), W1000: 1000-seed weight (g), COEF J: evaluated in seed; coefficient J = (length./width, COEF H: evaluated in the seed, Coefficient H = (thick./width.); and YLD: yield in g/plot. ^(2)^ The degrees of freedom were adjusted for the variable YLD (values subscribed in parentheses).

**Table 2:** Combined variance analysis for 23 agromorphological traits evaluated in the characterization study of common bean cultivars of the commercial group black in four environments in the state of Paraná in the rainy season of 2014/15 and dry season of 2015.

FV^1/^	Mean Square								Mean	CV(%)	>SMr/ <SMr
	Block/	Cultivars		Environment		CxE		Error			
	Environment	(C)		(E)							
PLL	1.058	1.176	**	93.061	**	0.44223	**	0.09289	6.23	4.89	2.43
PLW	0.555	0.564	**	8.103	**	0.23662	**	0.05964	4.83	5.06	2.61
PLI	0.002	0.006	**	3.305	**	0.00522	**	0.0016	1.29	3.1	5.47
CLL	1.779	1.83	**	43.54	**	0.53553	**	0.3145	9.37	5.99	1.99
CLW	1.149	1.007	**	19.69	**	0.25768	**	0.14893	7.12	5.42	2.99
CLI	0.001	0.01	**	0.058	**	0.00305	ns	0.00234	1.32	3.68	1.32
StL	477.254	531.635	**	38879.19	**	148.9056	**	79.06334	84.48	10.53	5.65
IFP	8.051	30.841	**	907.166	**	10.19187	**	4.4347	16.19	13	1.58
NN	1.391	8.61	**	59.798	**	1.7739	*	1.09563	14.33	7.3	2.54
PL	0.558	6.19	**	10.591	**	0.28984	**	0.13967	10.36	3.61	2.96
NSP	0.448	1.125	**	3.212	*	0.16299	ns	0.12617	6.21	5.72	1.24
LP	0.29	1.204	**	0.993	ns	0.10197	*	0.06978	6.76	3.91	1.73
NPP	91.735	62.198	**	1474.494	**	25.14098	**	14.92119	21.65	17.85	2.68
NSP	2149.489	1407.544	**	28636.17	**	686.3143	**	371.5215	103.62	18.6	2.26
StTh	1.589	1.48	**	8.90346	*	0.40876	**	0.23695	6.54	7.45	3.01
SL	0.122	1.521	**	8.66127	**	0.07669	ns	0.06325	10.34	2.43	5.48
SWth^(2)^	0.07	0.677	**	3.99963	**	0,09338^(28)^	ns	0,08138^(60)^	6.53	4.37	10.84
STh^(2)^	0.125	0.36	**	5.8179	**	0,08197^(30)^	ns	0,07612^(66)^	4.79	5.76	9.32
TSW	102.078	84.234	**	2442.32	**	34.39224	**	19.09978	23.44	18.64	2.58
W1000	473.183	4081.169	**	38436.96	**	498.8703	**	211.2958	225.07	6.46	1.92
COEF J	0.001	0.041	**	0.01887	**	0.00228	**	0.00131	1.58	2.28	4.43
COEF H	0.002	0.007	**	0.04836	**	0.00101	**	0.00043	0.73	2.82	3.55
YLD^(2)^	251503.2	75933.08	ns	1397689	*	51699,5^(35)^	ns	52672,97^(79)^	1785.2	32.14	12.92

*/** significant at 5% and 1% probability, respectively

ns = not significant, Test F, DF Blocks/Environment = 8, DF Cultivars = 19, DF Environment = 3, DF CxE = 57, DF Residue = 152, ^1/^PLL: primary leaf length (cm); PLW: primary leaf width (cm); PLI: primary leaf index (PLL/PLW); CLL: central leaflet length (cm); CLW: central leaflet width (cm); CLI: central leaf index (CLL/ CLW); StL: main stem length (cm); IFP: insertion height of the first pod (cm), NN: number of stem nodes, PL: pod length (cm); NSP: number of seeds per pod; LP: number of locules per pod; NPP: number of pods per plant; NSP: number of seeds per plant; StTh: main stem thickness (mm); SL: seed length; SWth: Seed width (mm); STh: seed thickness; TSW: total seed weight in the plant (g), W1000: 1000-seed weight (g), COEF J: evaluated in seed; coefficient J = (length./width, COEF H: evaluated in the seed, Coefficient H = (thick./width.); and YLD: yield in g/plot. ^(2)^ The degrees of freedom were adjusted for the variables SWth, STh and YLD (values subscribed in parentheses).

In case of GA interaction, [34] suggested that groupings based on only one year of cultivation can generate misleading information. Therefore, the evaluation of cultivars in different years and environments may be safer, and the larger the number of environments evaluated the better, regardless of the dissimilarity measure and clustering method being used.

**Fig 1:**
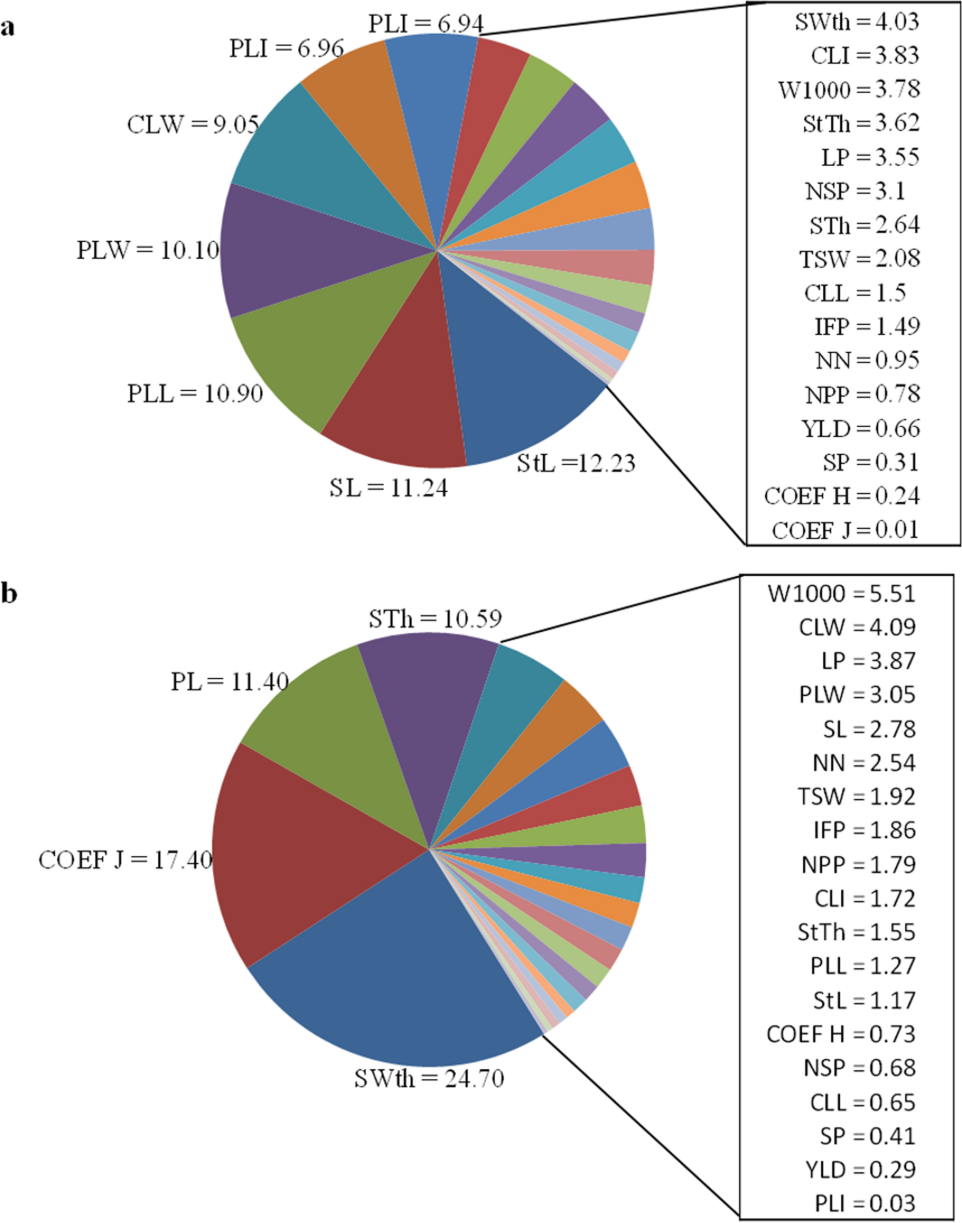
**Relative importance of 23 quantitative descriptors of carioca (a) and black (b) common bean grain for phenotypic divergence by the method of Singh (1981).** PLL: primary leaf length (cm); PLW: primary leaf width (cm); PLI: primary leaf index (PLL/PLW); CLL: central leaflet length (cm); CLW: central leaflet width (cm); CLI: central leaf index (CLL/ CLW); StL: main stem length (cm); IFP: insertion height of the first pod (cm), NN: number of stem nodes, PL: pod length (cm); NSP: number of seeds per pod; LP: number of locules per pod; NPP: number of pods per plant; NSP: number of seeds per plant; StTh: main stem thickness (mm); SL: seed length; SWth: Seed width (mm); STh: seed thickness; TSW: total seed weight in the plant (g), W1000: 1000-seed weight (g), COEF J: evaluated in seed; coefficient J = (length./width, COEF H: evaluated in the seed, Coefficient H = (thick./width.); and YLD: yield in g/plot.

The analysis of genetic divergence among cultivars of the commercial group carioca, according to the method proposed by [19], showed that the traits with highest relative importance were StL, SL, PLL, PLW, and CLW with 12.23, 11,24, 10.90, 10.10, and 9.05%, respectively (Fig 1). For the black group, the most important were SWth, COEF J, PL, and STh, with a relative importance of 24.70, 17.40, 11.40, and 10.59%, respectively. These results confirm the importance of distinct variables among the groups, such as those related to grain for the black group and to plant morphology for the carioca group.

The evaluation of the relative importance of traits makes it possible to discard traits that contribute least to the discrimination of the genotypes, reducing costs and labor in the following experiments. For example, in the black commercial group, the trait seed size has often been used to distinguish cultivars. In an evaluation of 27 common bean accessions. [35] found that seed size was the trait that most contributed to discriminate accessions. The results of this study showed a low discriminatory power of YLD in both groups, in spite of the commercial relevance of this trait. An explanation for this result is the low variation in productivity means, especially for the black group, in which no differences between cultivars were detected (Table 2). Similar results were reported by [36,37].

**Fig 2:**
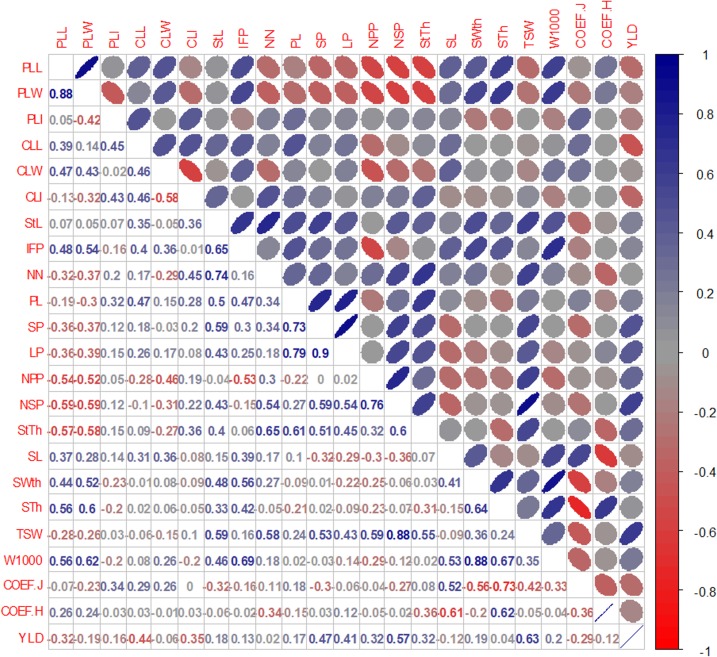
Estimates of Pearson’s correlation coefficient among 23 quantitative descriptors evaluated in 20 common bean genotypes of the carioca group. PLL: primary leaf length (cm); PLW: primary leaf width (cm); PLI: primary leaf index (PLL/PLW); CLL: central leaflet length (cm); CLW: central leaflet width (cm); CLI: central leaf index (CLL/ CLW); StL: main stem length (cm); IFP: insertion height of the first pod (cm), NN: number of stem nodes, PL: pod length (cm); NSP: number of seeds per pod; LP: number of locules per pod; NPP: number of pods per plant; NSP: number of seeds per plant; StTh: main stem thickness (mm); SL: seed length; SWth: Seed width (mm); STh: seed thickness; TSW: total seed weight in the plant (g), W1000: 1000-seed weight (g), COEF J: evaluated in seed; coefficient J = (length./width, COEF H: evaluated in the seed, Coefficient H = (thick./width.); and YLD: yield in g/plot.

**Fig 3:**
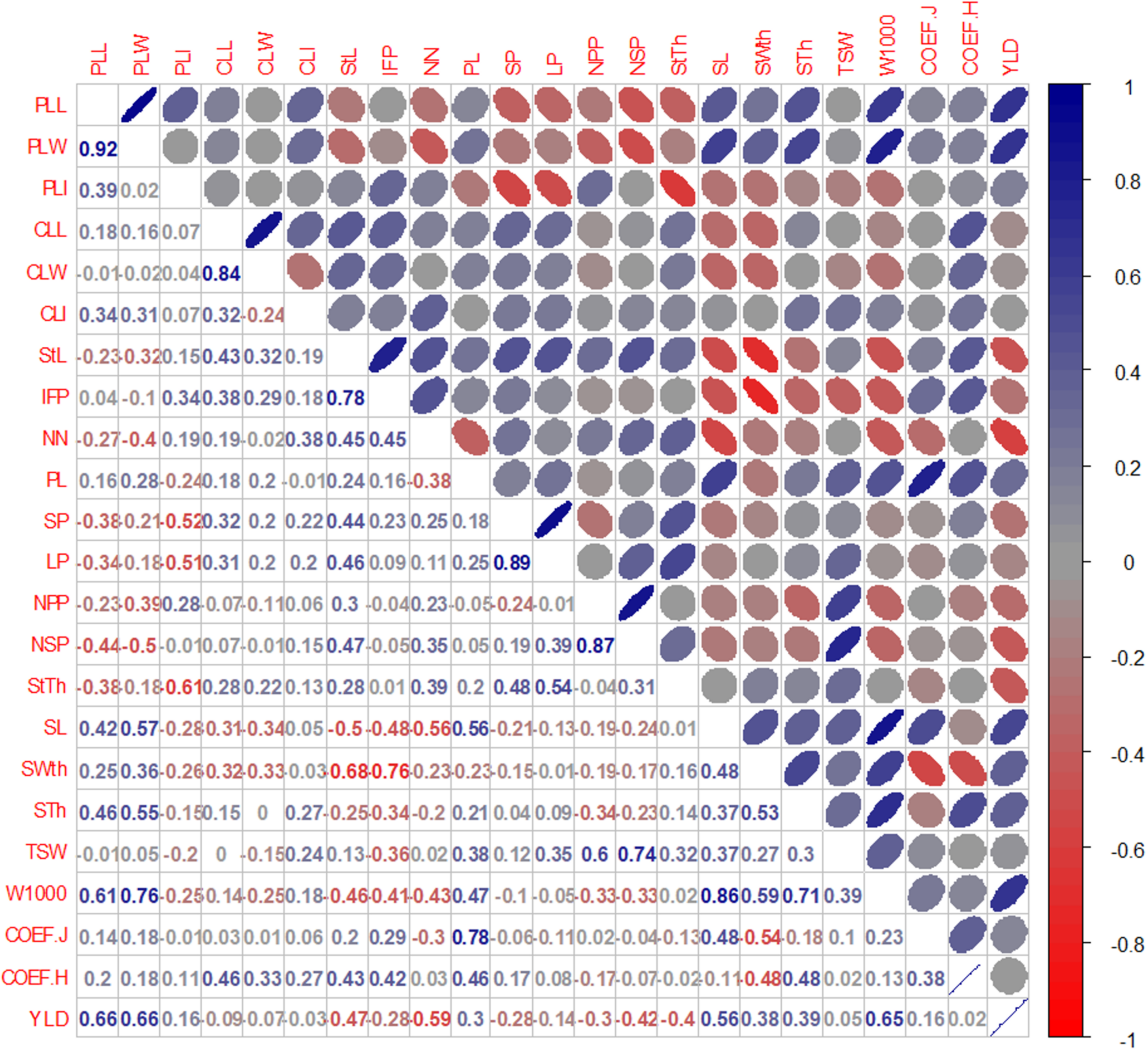
Estimates of Pearson’s correlation coefficient among 23 quantitative descriptors evaluated in 19 black common bean genotypes of the black group. PLL: primary leaf length (cm); PLW: primary leaf width (cm); PLI: primary leaf index (PLL/PLW); CLL: central leaflet length (cm); CLW: central leaflet width (cm); CLI: central leaf index (CLL/ CLW); StL: main stem length (cm); IFP: insertion height of the first pod (cm), NN: number of stem nodes, PL: pod length (cm); NSP: number of seeds per pod; LP: number of locules per pod; NPP: number of pods per plant; NSP: number of seeds per plant; StTh: main stem thickness (mm); SL: seed length; SWth: Seed width (mm); STh: seed thickness; TSW: total seed weight in the plant (g), W1000: 1000-seed weight (g), COEF J: evaluated in seed; coefficient J = (length./width, COEF H: evaluated in the seed, Coefficient H = (thick./width.); and YLD: yield in g/plot.

Results of Pearson’s correlation analysis revealed that for the carioca group only the variables CLL, NSP, NSP, and TSW (-0.44, 0.47, 0.57, and 0.63, respectively) were correlated with YLD, while for the black group, variables PLL, PLW, StL, NN, SL, and W1000 (0.66, 0.66, -0.47, -0.59, 0.56, and 0.65, respectively) correlated with YLD. These results indicated that the variables correlated distinctly with YLD in the two commercial groups (Figs 2 and 3). For the black group, variables related to shorter plants, and larger primary leaves and seeds are indicated to increase YLD, while for the carioca group a higher number of seeds per pod and, consequently, per plant is indicated. However, the variables related to YLD must be selected carefully, due to the correlation values (<0.70) and since the direct and indirect effects of these variables were not measured.

**Fig 4:**
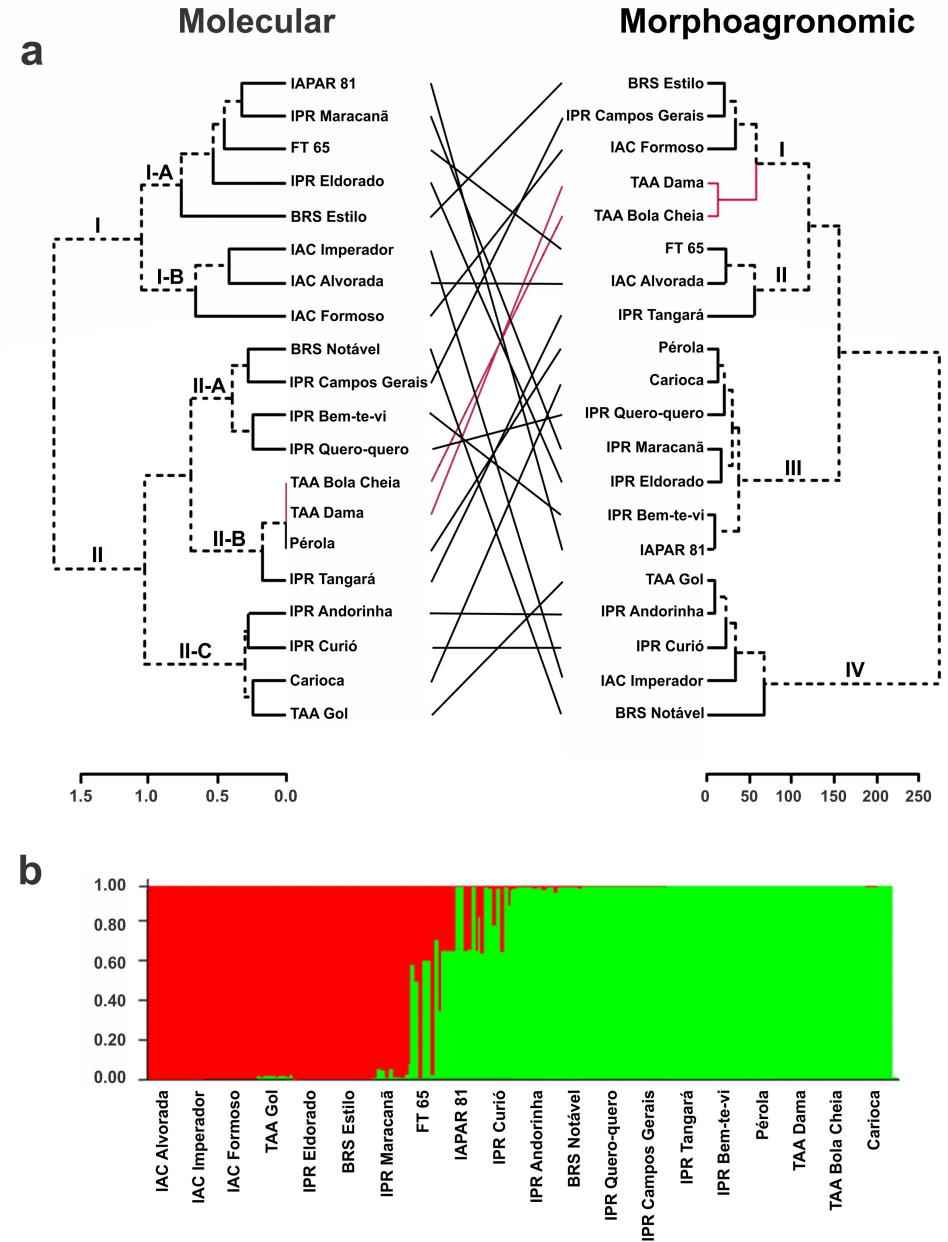
Grouping of 20 carioca common bean cultivars. (**a**) Dendrogram obtained by Ward’s method based on the dissimilarity matrix of molecular and morphoagronomic descriptors, and (**b**) analysis of the structure for molecular data (k = 2).

Using Ward's hierarchical clustering based on the distance of Mahalanobis, we observed the formation of four clusters in the carioca group (Fig 4). Cluster I contained five cultivars, which had the highest means in some production components (NSP, LP, NPP, NSP, and TSW) and, consequently, the highest productivity (S4 Table). Cluster II was associated with three cultivars with highest means in length and width of primary leaves and central leaflets (PLL, PLW, CLL, and CLW), higher StL and IFP, and also higher values of SL and SWth, resulting in higher W1000. However, this group had the lowest mean for NPP. Cluster III was the largest, consisting of seven cultivars, with intermediate values for most of the variables. Cluster IV consisted of five cultivars, with an early cycle and determinate growth habit. Although these cycle and size traits were not considered in the analyses, they were reflected in StL, which had a lower mean than in the other groups. The variables IFP, NN, PL and TSW were also lower for this group.

**Fig 5:**
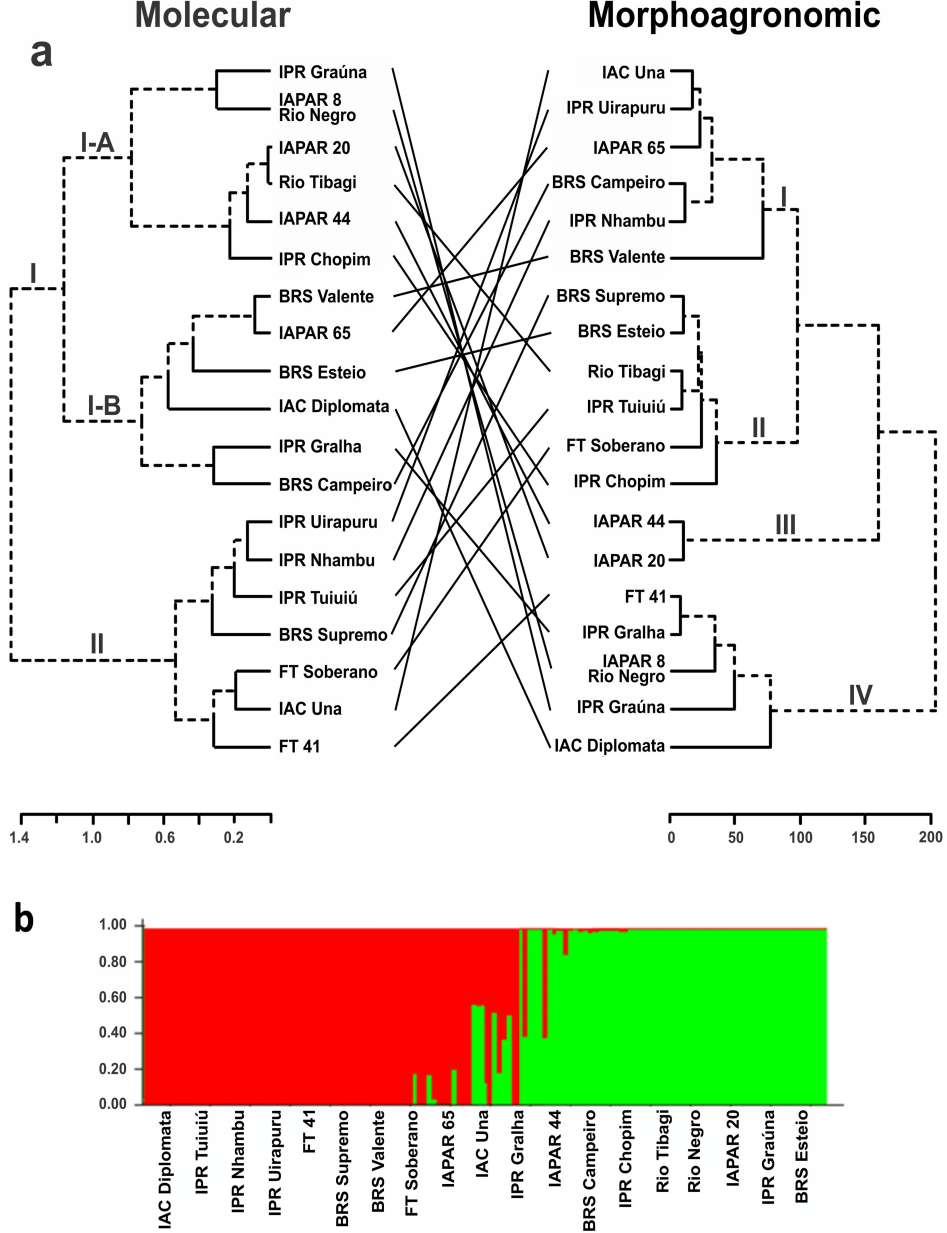
Grouping of 19 black common bean cultivars. (**a**) Dendrogram obtained by the Ward method based on the dissimilarity matrix of molecular and morphoagronomic descriptors, and (**b**) analysis of the structure for molecular data (k = 2).

Four distinct clusters were also observed in the black group (Fig 5). Cluster I comprised six cultivars, and in this group the means for the variables StL, IFP, NN, NSP, LP, and NSP were the lowest. On the other hand, the highest values ​​for the seed-related variables (SL, SWth, TSW, and W1000) were found in this group, resulting in the highest YLD mean (S5 Table). Cluster II, also containing six cultivars, had intermediate values for most of the variables, except for NSP and LP, for which the values were highest, and NPP and TSW with the lowest values. Cluster III consisted of only the cultivars IAPAR 44 and IAPAR 20, characterized by the high values of StL, NN and NSP, while W1000 and YLD had the lowest means. Cluster IV contained five cultivars with highest means for CLL, LFC, PL, NPP, and COEF J.

### Molecular characterization

Molecular analysis of 37 microsatellite loci identified a total of 116 alleles, of which 33 loci were polymorphic among the cultivars of the carioca group. In the black group, a total of 107 alleles were identified, with 30 polymorphic loci. Linkage disequilibrium was not detected in any of the loci. The number of alleles per locus (*A*) varied from 1 to 8, with a mean of 3.40 and 2.89 alleles per locus for the carioca and black groups, respectively (Table 3). The number of effective alleles (*Ae*) for each locus ranged from 1 to 4.81 and 1 to 3.69, respectively, for the carioca and black groups, while the number of null alleles (*An*) varied from 0 to 1 at different loci in both groups. The polymorphic information content (*PIC*) ranged from 0 to 0.76 (mean of 0.35) in the carioca group and from 0 to 0.69 (mean of 0.32) in the black group. The Shannon index (*I*) ranged from 0 to 1.75 and 0 to 1.44, with means of 0.69 and 0.65, respectively, for the carioca and black groups, indicating a moderate diversity level between cultivars for both groups.

**Table 3:** Genetic parameters for 37 microsatellite markers applied in 20 and 19 common bean cultivars of the carioca and black groups, respectively.

Primers	Genetic parameters^1/^									
	Carioca Group					Black Group				
	*A*	*Ae*	*An*	*PIC*	*I*	*A*	*Ae*	*An*	*PIC*	*I*
BM114	2	1.11	0.74	0.09	0.2	3	2.95	0.91	0.59	1.09
BM143	6	3.82	0.9	0.69	1.45	6	3.63	0.98	0.68	1.47
BM151	3	2.03	0.97	0.44	0.86	2	1.26	0.94	0.19	0.36
BM165	4	2.19	0.96	0.49	1.01	4	1.53	0.99	0.32	0.68
BM181	3	1.2	0.8	0.15	0.32	2	1.78	0.97	0.34	0.63
BM183	3	1.82	0.93	0.41	0.8	3	2.22	0.91	0.49	0.93
BM185	7	4.1	0.97	0.72	1.6	5	3.69	0.96	0.69	1.44
BM187	3	2.19	0.99	0.48	0.92	4	2.09	0.95	0.48	0.98
BM 201	5	2	0.99	0.44	0.91	4	1.99	1	0.43	0.87
BM202	2	1.98	0.99	0.37	0.69	2	1.99	1	0.37	0.69
BM209	4	1.82	0.95	0.4	0.82	3	1.88	0.94	0.38	0.73
BM210	8	4.81	0.98	0.76	1.75	4	2.77	0.92	0.58	1.17
BM212	2	1.4	0.97	0.24	0.46	1	1	0	0	0
GATS91	5	3.1	0.97	0.63	1.29	6	2.83	1	0.61	1.33
PVBR5	6	2.92	1	0.59	1.23	4	2.68	1	0.55	1.11
PVBR11	2	1.46	0.98	0.27	0.5	2	1.11	0.75	0.1	0.21
PVBR35	3	1.45	0.95	0.29	0.59	4	2.66	0.92	0.55	1.1
PVBR87	2	1.32	0.95	0.21	0.4	3	1.24	0.92	0.19	0.41
PVBR113	4	1.74	0.99	0.37	0.75	3	1.66	0.93	0.35	0.69
PVBR163	4	2.23	0.96	0.51	1.03	4	2.09	0.95	0.48	0.98
PVBR181	1	1	0	0	0	1	1	0	0	0
PVBR185	3	1.83	0.99	0.38	0.73	3	1.62	0.99	0.34	0.67
PVBR198	2	1.68	0.95	0.32	0.59	2	1.11	0.75	0.1	0.21
PVBR243	3	2.59	0.98	0.54	1.02	7	2.96	1	0.63	1.41
BMd10	2	1.58	0.88	0.3	0.56	2	1.87	1	0.36	0.66
BMd20	3	1.38	0.9	0.25	0.52	2	1.97	0.95	0.37	0.69
BMd25	1	1	0	0	0	1	1	0	0	0
BMd26	1	1	0	0	0	1	1	0	0	0
BMd33	3	1.36	0.93	0.25	0.52	2	1.11	0.75	0.1	0.21
BMd36	3	1.45	0.95	0.28	0.56	4	2.06	1	0.44	0.87
BMd40	3	2.19	0.99	0.47	0.9	2	1.78	1	0.34	0.63
BMd42	3	2.72	1	0.56	1.04	3	2.47	0.98	0.51	0.97
BMd45	2	1.58	0.99	0.3	0.56	2	1.05	0.56	0.05	0.12
BMd53	2	1.02	0.34	0.02	0.06	1	1	0	0	0
PVat003	1	1	0	0	0	1	1	0	0	0
PVat008	3	1.99	0.82	0.39	0.76	3	1.66	0.7	0.33	0.63
PVag001	2	1.18	0.87	0.14	0.29	1	1	0	0	0
Média	3.14	1.92	0.83	0.35	0.69	2.89	1.86	0.75	0.32	0.65

1/Number of alleles (A), number of effective alleles (Ae), number of null alleles (An), polymorphic information content (PIC) and Shannon diversity index (I).

Similar data were found by [38], in an evaluation of 60 cultivars of the carioca group using 70 SSR markers, where two to six alleles per locus were found, with a mean of 2.8 alleles per locus and PIC between 0.03 and 0.7 (mean of 0.47). The genetic diversity of common bean cultivars and accessions was evaluated by [39] using 16 SSR markers, and observed that the number of alleles varied from two to four (mean of 2.23 alleles per locus) and the PIC ranged from 0.11 to 0.51 (mean of 0.27). These results agree with those of [13]. In an evaluation of 10 microsatellite loci in 85 common bean accessions from a germplasm bank, the authors found a variation of 3–10 alleles, with a mean of 7 alleles per locus, and PIC values between 0.23 and 0.8. This greater variability was already expected, since germplasm banks contain greater diversity than the cultivars of a commercial group.

Among the cultivars analyzed, seven were considered pure in the carioca group (IPR Quero-quero, IPR Tangará, BRS Estilo, TAA Gol, TAA Dama, TAA Bola Cheia, and Carioca) and five in the black group (IPR Uirapuru, BRS Campeiro, BRS Esteio, BRS Supremo, and BRS Valente) (Table 4). The cultivars IPR Eldorado, IPR Tuiuiu, FT 41, and IAPAR 8—Rio Negro had only one heterozygous locus. Of the heterogeneous cultivars, IPR Campos Gerais, IPR Maracanã, BRS Notável, and FT 65 of the carioca group had the highest number of heterozygous loci or with different alleles, and cultivars IAPAR 65, IPR Graúna, IPR Gralha, IAC Una and FT Soberano in the black group (Table 4).

**Table 4:** Genetic parameters found in 20 and 19 common bean cultivars of the carioca and black groups, respectively, using 37 microsatellite markers.

Carioca Group						Black Group					
Cultivar	Purity of cultivars		Genetic Parameters^1/^			Cultivar	Pureza de cultivares		Genetic Parameters		
	N^o^ of heterozygotes loci	N^o^ of loci with different alleles	*PP(%)*	*A*	*Ae*		N^o^ of heterozygotes loci	N^o^ of loci with different alleles	*PP(%)*	*A*	*Ae*
IAPAR 81	-	3	8.11	1.08	1.06	IAPAR 8- Rio Negro	1	-	2.70	1.03	1.02
IPR Eldorado	1	-	2.70	1.03	1.00	IAPAR 20	9	2	29.73	1.42	1.13
IPR Tangará	-	-	0.00	1.00	1.00	IAPAR 44	3	4	18.92	1.23	1.10
IPR Campos Gerais	-	7	18.90	1.19	1.16	IAPAR 65	1	12	35.14	1.43	1.35
IPR Curió	2	3	13.51	1.19	1.11	Rio Tibagi	2	2	10.81	1.14	1.04
IPR Andorinha	-	4	10.81	1.14	1.09	IPR Uirapuru	-	-	0.00	1.00	1.00
IPR Maracanã	1	9	27.00	1.38	1.26	IPR Chopim	-	3	8.11	1.08	1.08
IPR Bem-te-vi	-	3	8.11	1.08	1.07	IPR Graúna	-	6	16.22	1.16	1.11
IPR Quero-quero	-	-	0.00	1.00	1.00	IPR Gralha	2	8	27.03	1.32	1.23
Pérola	2	-	5.41	1.05	1.02	IPR Tuiuiú	1	-	2.70	1.03	1.02
BRS Estilo	-	-	0.00	1.00	1.00	IPR Nhambu	1	4	13.51	1.14	1.11
BRS Notável	4	6	27.00	1.46	1.30	BRS Valente	-	-	0.00	1.00	1.00
Carioca	-	-	0.00	1.00	1.00	BRS Campeiro	-	-	0.00	1.00	1.00
IAC Alvorada	3	1	10.81	1.11	1.04	BRS Supremo	-	-	0.00	1.00	1.00
IAC Formoso	1	2	8.11	1.08	1.04	BRS Esteio	-	-	0.00	1.00	1.00
IAC Imperador	-	3	8.11	1.08	1.04	IAC Una	7	7	37.84	1.43	1.35
FT-65	10	6	43.2	1.73	1.48	IAC Diplomata	1	2	8.11	1.08	1.06
TAA Bola Cheia	-	-	0.00	1.00	1.00	FT Soberano	-	9	24.32	1.27	1.19
TAA Gol	-	-	0.00	1.00	1.00	FT 41	1	-	2.70	1.03	1.02
TAA Dama	-	-	0.00	1.00	1.00						
Média	-	-	9.59	1.13	1.08	Média	-	-	12.52	1.15	1.09

^1/^Percentage of polymorphic loci (PP (%)), mean number of alleles per locus (A) and mean number of effective alleles (Ae).

The presence of homozygous loci for different alleles may be related to the occurrence of crosses (gene flow) with other common bean plants from the surrounding fields or contaminating plants in the experimental field, followed by the fixation of the exogenous allele in the next selfing generations. In contrast, the heterozygous loci can be attributed to the residual heterozygosity present in some plants, even after several generations of selfing, or it may be related to recent natural crosses with other distinct genotypes [40]. This may have occurred with the cultivars FT-65 and IAPAR 20, which after long standing cultivation may have reached a high level of heterozygosity.

Although outcrosssing responds for a small percentage of the reproduction of common bean, gene flow occurs among cultivars. In the estimation of gene flow among bean cultivars of the carioca group, an average of 77% of non-migrants was observed in each cultivar and an average of 23% of migrants (approximately 1,2% of migrants from each cultivar). Similar results were observed for the black group. These values were probably found due to the close genetic base among bean cultivars, where most of them share characteristics derived from common ancestors. Among the commercial groups, carioca and black, no gene flow was detected.

In the molecular variance analysis (AMOVA) carried out among the commercial groups, the index of fixation (Fst) was high (0.912), indicating a high genetic differentiation, being that between the groups the percentage of variation is of 29%. However, the greater genetic variability is found among cultivars within each commercial group (62.08%). Furthermore only 8% of the variability is found within cultivars, as expected of autogamous species (Table 5).

**Table 5:** Analysis of molecular variance (AMOVA) among 20 common beans cultivars from the carioca group and 19 of the black group.

Source of variation	Degrees of freedom	Sum of squares	Variance componentes	Percentage of variation
Among commercial groups	1	1095.575	2.98865	29.11*
Among cultivars within comercial groups	36	4091.065	6.37417	62.08*
Within cultivars	634	573.689	0.90487	8.81*
Total	671	5760.329	10.26769	
Fixation index (*Fst*)	0.91187*			

* p>0,05; (Significance test performed through 1023 permutations)

Twenty-four private alleles were identified in 16 of the loci analyzed in the carioca group, while in the black group, 22 alleles were found, also distributed in 16 of the analyzed loci. Among the cultivars of the carioca group, the highest numbers of private alleles was observed in cultivars IPR Curió and BRS Notável, with four private alleles each, and IPR Maracanã, with six private alleles. In the black group, cultivar IAC Diplomata contained the highest number (eight) of private alleles (Table 6). Private alleles facilitate cultivar identification, allowing a fast and efficient detection of test cultivars.

**Table 6:** Number of private alleles (*Ap*) found in common bean cultivars of the carioca and black groups and their respective loci and base pair sizes (bp).

Cultivar	*Ap*	Locus—bp
Carioca Group		
IPR Campos Gerais	2	BM 201–125; BM209-118
IPR Curió	4	BM185-107; BM210-174; PVBR5-214; PVat008-178
IPR Andorinha	1	BM114-266
IPR Maracanã	6	BM 201–117; BM185-119; BM210-184; PVBR163-243; BM143-154; BMd53-123
BRS Estilo	3	PVBR113-107; BM201-115; GATs91-248
BRS Notável	4	PVBR5-197; PVBR5-195; PVBR185-164; GATs91-272
FT-65	3	PVBR113-134; BM181-205; BMd36-191
TAA Gol	1	BM165-202
Black Group		
IAPAR 8 –Rio Negro	2	PVBR11-195; PVBR5-207
IPR Graúna	1	BM201-115;
IPR Gralha	2	PVBR243-260; BMd36-179
IPR Nhambu	1	PVBR243-239
BRS Campeiro	2	PVBR113-107; PVBR87-172
BRS Supremo	2	PVBR163-250; BM187-203;
BRS Esteio	2	PVBR243-258; PVBR185-164
IAC Diplomata	8	BM201-117; PVBR87-125; BM165-190; PVBR35-260; PVBR243-252; BMd36-187; GATs91-262; BMd45-143
FT Soberano	1	PVBR198-240
FT 41	1	BMd33-123

In the carioca group, the analysis of locus BM 201 detected three cultivars (IPR Maracanã, IPR Campos Gerais and BRS Estilo), while with the other loci containing private alleles it was possible to distinguish one or two cultivars. However, the cultivars were only distinguishable when considering the loci together. In the black group, the set of analyzed loci was able to distinguish all the cultivars. The private alleles make it possible to easily identify the cultivars IAPAR 8 –Rio Negro, IPR Graúna, IPR Gralha, IPR Nhambu, BRS Campeiro, BRS Supremo, BRS Esteio, IAC Diplomata FT Soberano, and FT 41 in the black group. Locus PVBR 243 had the highest number of private alleles, allowing the identification of the cultivars IPR Gralha, IPR Nhambu, BRS Esteio, and BRS Diplomata.

In an analysis of 114 common bean genotypes, including 50 commercial cultivars of public institutions in Brazil and other countries, and 64 lines used in breeding programs, [16] found 35 private alleles in 15 analyzed microsatellite loci and observed that more recent cultivars have a greater number of private alleles. These results possibly reflect the incorporation of new genotypes in the breeding programs, responding to the demand for cultivars with different traits.

In Ward’s hierarchical clustering on the basis of Nei’s distance [24], it was possible to observe the formation of two clusters in the two groups, carioca and black. These results were supported by Bayesian analysis for the number of clusters (k), which identified k = 2 (Figs 4 and 5). In the carioca group, the clusters generated by Ward and Structure coincided, except for cultivar TAA Gol and IAPAR 81 that were allocated in distinct clusters when comparing the groups. By molecular analysis it was not possible to differentiate the cultivars Pérola, TAA Dama and TAA Bola Cheia, which had the same amplification pattern for all loci. The cultivars of the IAC breeding program (IAC Imperador, IAC Alvorada and IAC Formoso) were grouped in the same cluster, while the IAPAR cultivars were distributed in both clusters (Fig 4).

For the black group, similarity was also observed between Ward’s and Structure clustering, except for the cultivars BRS Valente, IAC Diplomata, IAPAR 65, and IPR Gralha that were allocated in distinct clusters when comparing the groups (Fig 5). Cluster I was the most numerous, and was divided into two sub-clusters (I-A and I-B). In I-A, cultivars IPR Graúna, IAPAR 8—Rio Negro, IAPAR 20, Rio Tibagi, IAPAR 44, and IPR Chopim were allocated, most of which are descendants of cultivar Rio Tibagi, except for IPR Chopim (S2 Table). Sub-cluster I-B was composed of the cultivars of the breeding programs of Embrapa, IAPAR and IAC. Cluster II also comprised cultivars of several breeding programs.

The correlation between the morphoagronomic and molecular data matrices for the carioca and black beans group was not significant (r = -0.02 and -0.04, respectively). The same occurred in other studies, in which no correlation between morphoagronomic and molecular data was detected [18,41–43]. The absence of correlation is probably due to the fact that the markers selected in this study were not related to the morphoagronomic traits evaluated. Microsatellites are present in both coding and non-coding regions and are therefore not necessarily linked to the expression of morphological traits [35]. In addition, many of the evaluated morphoagronomic traits are controlled by a high number of genes, being easily influenced by the environment.

With a view to cultivar protection and identification, the molecular markers were more efficient. For the morphoagronomic traits required by the official descriptors, most of which are qualitative, and the cultivars had little or no variability. Therefore, it is often difficult to visualize differences for some traits, restricting the discriminating power of cultivars, mainly in species with a narrow genetic basis. The molecular markers however were able to differentiate all cultivars, except for TAA Dama and TAA Bola Cheia.

Discrimination by molecular markers can be used as complementary to the DHS test, since the results are reproducible, consistent and not influenced by the environment or the reproductive stage of the plant [16]. High Resolution Melting analysis (HRM), also could be used as an alternative technique to investigate microsatellites. This is a method that measures the rate of dissociation of double stranded DNA to single stranded DNA and with the progress of the technique, allowing the use of HRM for genotyping (SNPs, SSR markers) and for quantification of adulterations, therefore the method can be used in analysis for authenticity testing and quantitative detection of bean crops [44].

Molecular descriptors are already accepted for cultivar characterization by the United States Department of Agriculture (USDA), whereas in Brazil the molecular analyses are not yet recognized as official methods for registration and protection of common bean cultivars [45]. To date, molecular markers for cultivar protection can only be used for sugarcane in Brazil. However, their use is optional and complementary to the morphoagronomic descriptors.

## Supporting information

S1 Table Cultivars of the commercial group carioca, genealogy, institution and year of registration in the national register of cultivar of the Ministry of Agriculture, Livestock and Supply (RNC/MAPA)–Brazil. (DOCX)

S2 Table Cultivars of the commercial group black, genealogy, institution and year of registration in the national register of cultivar of the Ministry of Agriculture, Livestock and Supply (RNC/MAPA)–Brazil. (DOCX)

S3 Table Microsatellite primers used for characterization of 20 and 19 bean cultivars of the commercial groups carioca and black, respectively. ^1^Genomic distribution by the cromossomes.
(DOCX)

S4 Table Mean and standard deviation of the 23 agromorphological traits for the groups formed by the Ward method from the Mahalanobis distance for cultivars of the commercial group carioca. ^1/^PLL: primary leaf length (cm); PLW: primary leaf width (cm); PLI: primary leaf index (PLL/PLW); CLL: central leaflet length (cm); CLW: central leaflet width (cm); CLI: central leaf index (CLL/ CLW); StL: main stem length (cm); IFP: insertion height of the first pod (cm), NN: number of stem nodes, PL: pod length (cm); NSP: number of seeds per pod; LP: number of locules per pod; NPP: number of pods per plant; NSP: number of seeds per plant; StTh: main stem thickness (mm); SL: seed length; SWth: Seed width (mm); STh: seed thickness; TSW: total seed weight in the plant (g), W1000: 1000-seed weight (g), COEF J: evaluated in seed; coefficient J = (length./width, COEF H: evaluated in the seed, Coefficient H = (thick./width.); and YLD: yield in g/plot.
(DOCX)

S5 Table Mean and standard deviation of the 23 agromorphological traits for the groups formed by the Ward method from the Mahalanobis distance for cultivars of the commercial group black. ^1/^PLL: primary leaf length (cm); PLW: primary leaf width (cm); PLI: primary leaf index (PLL/PLW); CLL: central leaflet length (cm); CLW: central leaflet width (cm); CLI: central leaf index (CLL/ CLW); StL: main stem length (cm); IFP: insertion height of the first pod (cm), NN: number of stem nodes, PL: pod length (cm); NSP: number of seeds per pod; LP: number of locules per pod; NPP: number of pods per plant; NSP: number of seeds per plant; StTh: main stem thickness (mm); SL: seed length; SWth: Seed width (mm); STh: seed thickness; TSW: total seed weight in the plant (g), W1000: 1000-seed weight (g), COEF J: evaluated in seed; coefficient J = (length./width, COEF H: evaluated in the seed, Coefficient H = (thick./width.); and YLD: yield in g/plot.
(DOCX)
