# Supplementary material for: Distinctness of Brazilian common bean cultivars with carioca and black grain by means of morphoagronomic and molecular descriptors
Source: PLoS One. 2017 Nov 30;12(11):e0188798. doi: 10.1371/journal.pone.0188798 (PMC5708700; doi:10.1371/journal.pone.0188798)
Supplement: S1 Table — (DOCX) [file pone.0188798.s001.docx]

**S1 Table.** Cultivars of the commercial group carioca, genealogy, institution and year of registration in the national register of cultivar of the Ministry of Agriculture, Livestock and Supply (RNC/MAPA) – Brazil.

| **Cultivars** | **Genealogy** | **Instittution** | **Year of registration (RNC/MAPA)** |
| --- | --- | --- | --- |
| IAPAR 81 | A248/ EMP117 /4/ BAT 93 /2/ carioca sel. 99 / Great Northern Nebraska 1 # 27 /3/ Sel. Aroana | IAPAR | 1998 |
| IPR Eldorado | RM8454-21-1-cm / IAPAR 14 | IAPAR | 2007 |
| IPR Tangará | LP 95-92 / Pérola | IAPAR | 2008 |
| IPR Campos Gerais | IAPAR 80/Campeão 2 | IAPAR | 2011 |
| IPR Curió | IAPAR 81/linhagem Carioca 1070 | IAPAR | 2013 |
| IPR Andorinha | SEL37-20 (Irmã da IPR 139) /IPR Colibri | IAPAR | 2013 |
| IPR Maracanã | MD 632/IAPAR BAC 32 | IAPAR | 2013 |
| IPR Bem-te-vi | BAT 93 /2/ Carioca Sel. 99/Great Northern Nebraska 1 sel. 27 /3/ Sel. Aruana / IAPAR 31 /4/ Campeão 1 | IAPAR | 2014 |
| IPR Quero-quero | LP02-21 / LP02-22. | IAPAR | 2014 |
| Pérola | Seleção na cultivar Aporé | Embrapa | 1998 |
| BRS Estilo | EMP250 /4/ A769 /// A429 / XAN 252 // V8025 / Pinto VI 114 | Embrapa | 2009 |
| BRS Notável | A 769 /4/ A 774 /// A 429 / XAN 252 // V 8025 / G 4449 /// WAF 2 /A55 // GN 31 / XAN 170 | Embrapa | 2013 |
| Carioca | Seleção de agricultores | IAC | 1998 |
| IAC Alvorada | IAC Carioca Pyatã/A686 /2/ IAC Maravilha /G2338 /3/ IAC Maravilha/And277 /4/ L317-1 | IAC | 2007 |
| IAC Formoso | Vax1 / IAC - Carioca Aruã /2/ IAC - Carioca Akytã / IAPAR 14 /3/ A686 | IAC | 2010 |
| IAC Imperador | IAC Carioca Eté / Carioca Precoce /2/ IAC Carioca Eté /3/ Feijão 60 Dias | IAC | 2012 |
| FT65 | – | FT sementes | 2008 |
| TAA Bola Cheia | WB-SR9903 / IAPAR 31 | TAA | 2009 |
| TAA Gol | – | TAA | 2013 |
| TAA Dama | – | TAA | 2013 |
