# Supplementary material for: Distinctness of Brazilian common bean cultivars with carioca and black grain by means of morphoagronomic and molecular descriptors
Source: PLoS One. 2017 Nov 30;12(11):e0188798. doi: 10.1371/journal.pone.0188798 (PMC5708700; doi:10.1371/journal.pone.0188798)
Supplement: S2 Table — (DOCX) [file pone.0188798.s002.docx]

**S2 Table.** Cultivars of the commercial group black, genealogy, institution and year of registration in the national register of cultivar of the Ministry of Agriculture, Livestock and Supply (RNC/MAPA) – Brazil.

| **Cultivars** | **Genealogy** | **Instittution** | **Year of registration (RNC/MAPA)** | |
| --- | --- | --- | --- | --- |
| IAPAR 8 - Rio Negro | PI307822 / PI310797 // Turrialba51052 / Cornell49-242 /3/ Rio Tibagi / Cornell 49-242 | IAPAR | 1998 | |
| IAPAR 20 | Rio Tibagi*^3^ / Cornell49-242 | IAPAR | 1998 | |
| IAPAR 44 | IAPAR BAC 2 /RAI 12 // Rio Tibagi*^1^ / Cornell 49-242 | IAPAR | | 1998 |
| IAPAR 65 | IAPAR BAC 38 / MD648 (Seleção DOR 0014-M-M-M-C) // GF3721 / IAPAR BAC 25 | IAPAR | | 1998 |
| Rio Tibagi | Introduzida do Instituto Interamericano de Ciências Agrícolas – Costa Rica | IAPAR | | 1998 |
| IPR Uirapuru | IAPAR BAC 29 / PR1711 /3/ NEP2 /2/ PUEBLA 173 / Icapijao | IAPAR | | 2000 |
| IPR Chopim | F7 (BAT 93 /2/ Carioca sel. 99 / Great Northern Nebraska 1 sel. # 27 / 3 / Sel. Aroana /4/ A176 / A259) / NEP 2 | IAPAR | | 2000 |
| IPR Graúna | EP173 / 2 / Rio Iguaçu / Great Northern Nebraska 1 sel. # 27 / 3 / Rio Tibagi / Cornell 49242 /4/ IAPAR BAC 25 /5/ IAPAR BAC 26 | IAPAR | | 2002 |
| IPR Gralha | IAPAR 14 / IAPAR 31 // EMPASC 201 / IAPAR 20 | IAPAR | | 2007 |
| IPR Tuiuiú | IPR Uirapuru / Xamego | IAPAR | | 2010 |
| IPR Nhambu | FT Tarumã / IAC Una // IPR Uirapuru | IAPAR | | 2014 |
| BRS Valente | Engopa 201- Ouro//Ônix/linhagem NA 512586 | Embrapa | | 2001 |
| BRS Campeiro | Radiação Gama na Cultivar Corrente | Embrapa | | 2004 |
| BRS Supremo | W22-34 / VAN163 | Embrapa | | 2007 |
| BRS Esteio | FT85-113 / POT 51 | Embrapa | | 2012 |
| IAC Una | DOR41 / H 1178-100 | IAC | | 1998 |
| IAC Diplomata | IAC Carioca Pyatã/A686 /2/ IAC Maravilha/G2338 /3/ IAC Maravilha/And277 /4/ L317-1 | IAC | | 2007 |
| FT Soberano | – | FT sementes | | 2001 |
| FT 41 | – | FT sementes | | 2009 |
