# Supplementary material for: Distinctness of Brazilian common bean cultivars with carioca and black grain by means of morphoagronomic and molecular descriptors
Source: PLoS One. 2017 Nov 30;12(11):e0188798. doi: 10.1371/journal.pone.0188798 (PMC5708700; doi:10.1371/journal.pone.0188798)
Supplement: S3 Table — 1Genomic distribution by the cromossomes. (DOCX) [file pone.0188798.s003.docx]

**S3 Table.** Microsatellite primers used for characterization of 20 and 19 bean cultivars of the commercial groups carioca and black, respectively.

| **Primers** |  | **Sequence** | **Fragment**  **size** | **Motif** | **Crm^1^** | **References** |
| --- | --- | --- | --- | --- | --- | --- |
| BM114 | D | AGCCTGGTGAAATGCTCATAG | 262 - 266 | (TA)8(GT)10 | 9 | GAITÁN-SOLÍS et al., 2002 |
|  | R | CATGCTTGTTGCCTAACTCTCT |  |  |  |  |
| BM143 | D | GGGAAATGAACAGAGGAAA | 81 - 168 | (GA)35 | 9 | GAITÁN-SOLÍS et al., 2002 |
|  | R | ATGTTGGGAACTTTTAGTGTG |  |  |  |  |
| BM151 | D | CACAACAAGAAAGACCTCCT | 159 - 163 | (TC)14 | 8 | GAITÁN-SOLÍS et al., 2002 |
|  | R | TTATGTATTAGACCACATTACTTCC |  |  |  |  |
| BM165 | D | TCAAATCCCACACATGATCG | 184 - 202 | (TA)3(CA)9 | 3 | GAITÁN-SOLÍS et al., 2002 |
|  | R | TTCTTTCATTCATATTATTCCGTTCA |  |  |  |  |
| BM181 | D | ATGCTGCGAGTTAATGATCG | 197 - 205 | (CT)17 | 5 | GAITÁN-SOLÍS et al., 2002 |
|  | R | TGAGGAGCAAACAGATGAGG |  |  |  |  |
| BM183 | D | CTCAAATCTATTCACTGGTCAGC | 158 - 162 | (TC)14 | 4 | GAITÁN-SOLÍS et al., 2002 |
|  | R | TCTTACAGCCTTGCAGACATC |  |  |  |  |
| BM185 | D | AAGGAGGTTTCTACCTAATTCC | 107 - 131 | (CT)12 | 4 | GAITÁN-SOLÍS et al., 2002 |
|  | R | AAAGCAGGGATGTAGTTGC |  |  |  |  |
| BM187 | D | TTTCTCCAACTCACTCCTTTCC | 177 - 203 | (CT)10T(CT)14 | 1 | GAITÁN-SOLÍS et al., 2002 |
|  | R | TGTGTTTGTGTTCCGAATTATGA |  |  |  |  |
| BM201 | D | TGGTGCTACAGACTTGATGG | 115 - 129 | (GA)15 | 2 | GAITÁN-SOLÍS et al., 2002 |
|  | R | TGTCACCTCTCTCCTCCAAT |  |  |  |  |
| BM202 | D | ATGCGAAAGAGGAACAATCG | 165 - 167 | (GA)9GT(GA)4 | - | GAITÁN-SOLÍS et al., 2002 |
|  | R | CCTTTACCCACACGCCTTC |  |  |  |  |
| BM209 | D | CAACCAATGAATGCTGACAATG | 84 - 122 | (TA)4(TG)16 | 7 | GAITÁN-SOLÍS et al., 2002 |
|  | R | CAATTTCTTGATTGAAAGGCAAT |  |  |  |  |
| BM210 | D | ACCACTGCAATCCTCATCTTTG | 174 - 200 | (CT)15 | 4 | GAITÁN-SOLÍS et al., 2002 |
|  | R | CCCTCATCCTCCATTCTTATCG |  |  |  |  |
| BM212 | D | AGGAAGGGATCCAAAGTCACTC | 209 - 219 | (CA)13 | 8 | GAITÁN-SOLÍS et al., 2002 |
|  | R | TGAACTTTCAGGTATTGATGAATGAAG |  |  |  |  |
| GATS91 | D | GAGTGCGGAAGCGAGTAGAG | 248 - 272 | (GA)17 | 9 | GAITÁN-SOLÍS et al., 2002 |
|  | R | TCCGTGTTCCTCTGTCTGTG |  |  |  |  |
| PVBR5 | D | ATTAGACGCTGATGACAGAG | 181 - 214 | (GA)22 | 1 | BUSO et al., 2006 |
|  | R | AGCAGAATCCTTTGAGTGTG |  |  |  |  |
| PVBR11 | D | AAACTCAAAGTCGTTGTTCC | 195 - 200 | (TC)8(GT)4 | 9 | BUSO et al., 2006 |
|  | R | CCACTGACTCTAGCTCCTCC |  |  |  |  |
| PVBR35 | D | TCTACGCGTTCCCTCTGTCT | 219 - 260 | (TC)10 | 4 | GRISI et al., 2007 |
|  | R | AGTGGATGTGTGGGAAAAGC |  |  |  |  |
| PVBR87 | D | CTCATTGCGTCTACCAGTGC | 172 - 176 | (GA)16 | 5 | GRISI et al., 2007 |
|  | R | CCTAGGTTCCGCAGCATGT |  |  |  |  |
| PVBR113 | D | TGCATTCTTCCTCCCATCTT | 99 - 134 | (TC)12 | 10 | GRISI et al., 2007 |
|  | R | TTGATTTGATTTGATCAGTGGTG |  |  |  |  |
| PVBR163 | D | TGAGAGTGGAGAAGGAGAGAGA | 224 - 250 | (AG)16 | 1 | GRISI et al., 2007 |
|  | R | TGACAACACTGCAAACACCA |  |  |  |  |
| PVBR181 | D | AGGGAAAAGTGGGAAAGGAA | 226 | (AG)11 | 8 | GRISI et al., 2007 |
|  | R | CCACAGCTGATGAAAAGCAA |  |  |  |  |
| PVBR185 | D | TGGTAAAGCAAAAACGATGG | 164 - 170 | (TC)11 | 8 | GRISI et al., 2007 |
|  | R | GACAGAAGAGTGAGGGTGTGAA |  |  |  |  |
| PVBR198 | D | GCAAGGAAGCCATAGACCAC | 230 - 240 | (AG)10 | 1 | GRISI et al., 2007 |
|  | R | AGAAGCCCAGTCCAAAGACA |  |  |  |  |
| PVBR243 | D | TCTGTCTGTGTATAAACACCGTTTT | 237 - 256 | (CT)20 | 9 | GRISI et al., 2007 |
|  | R | TGCGGAAGCGAGTATAGAGG |  |  |  |  |
| BMd-10 | D | GCTCACGTACGAGTTGAATCTCAG | 154 - 156 | (GA)8 | 1 | BLAIR et al.,2003 |
|  | R | ATCTGAGAGCAGCGACATGGTAG |  |  |  |  |
| BMd-20 | D | GTTGCCACCGGTGATAATCT | 122 - 142 | (TA)5 | 5 | BLAIR et al.,2003 |
|  | R | GTGAGGCAAGAAGCCTTCAA |  |  |  |  |
| BMd-25 | D | GCAGATCGCCTACTCACAAA | 128 | (GAT)6 | 8 | BLAIR et al.,2003 |
|  | R | CGTTGACGAGAAGCATCAAG |  |  |  |  |
| BMd-26 | D | CTTGCCTTGTGCTTCCTTCT | 156 | (GAT)6 | 4 | BLAIR et al.,2003 |
|  | R | TCCATTCCCAACCAAGTTTC |  |  |  |  |
| BMd-33 | D | TACGCTGTGATGCATGGTTT | 110 - 123 | (ATT)9 | 6 | BLAIR et al.,2003 |
|  | R | CCTGAAAGTGCAGAGTGGTG |  |  |  |  |
| BMd-36 | D | CATAACATCGAAGCCTCACAGT | 179 - 191 | (TA)8 | 3 | BLAIR et al.,2003 |
|  | R | ACGTGCGTACGAATACTCAGTC |  |  |  |  |
| BMd-40 | D | AACCTTCTTGCGCTGATCTC | 210 - 214 | (AT)6 | 7 | BLAIR et al.,2003 |
|  | R | TAGTGGCCATTCCTCGATCT |  |  |  |  |
| BMd-42 | D | TCATAGAAGATTTGTGGAAGCA | 164 - 174 | (AT)5 | 8 | BLAIR et al.,2003 |
|  | R | TGAGACACGTACGAGGCTGTAT |  |  |  |  |
| BMd-45 | D | GGTTGGGAAGCCTCATACAG | 104 - 143 | (AG)5 | 1 | BLAIR et al.,2003 |
|  | R | ATCTTCGACCCACCTTGCT |  |  |  |  |
| BMd-53 | D | TGCTGACCAAGGAAATTCAG | 120-123 | (GTA)5 | 5 | BLAIR et al.,2003 |
|  | R | GGAGGAGGCTTAAGCACAAA |  |  |  |  |
| PVat008 | D | AGTCGCCATAGTTGAAATTTAGGTG | 178 - 188 | (AT)9 | 3 | YU et al.,2000 |
|  | R | CTTATTAAAACGTGAGCATATGTATCATTC |  |  |  |  |
| PVat003 | D | ACCTAGAGCCTAATCCTTCTGCGT | 148 | (AT)6 | 4 | YU et al.,2000 |
|  | R | GAATGTGAATATCAGAAAGCAAATGG |  |  |  |  |
| PVag001 | D | CAATCCTCTCTCTCTCATTTCCAATC | 162 - 168 | (GA)11 | 11 | YU et al.,2000 |
|  | R | GACCTTGAAGTCGGTGTCGTTT |  |  |  |  |

^1^ Genomic distribution by the cromossomes
