# Supplementary material for: Distinctness of Brazilian common bean cultivars with carioca and black grain by means of morphoagronomic and molecular descriptors
Source: PLoS One. 2017 Nov 30;12(11):e0188798. doi: 10.1371/journal.pone.0188798 (PMC5708700; doi:10.1371/journal.pone.0188798)
Supplement: S4 Table — 1/PLL: primary leaf length (cm); PLW: primary leaf width (cm); PLI: primary leaf index (PLL/PLW); CLL: central leaflet length (cm); CLW: central leaflet width (cm); CLI: central leaf index (CLL/ CLW); StL: main stem length (cm); IFP: insertion height of the first pod (cm), NN: number of stem nodes, PL: pod length (cm); NSP: number of seeds per pod; LP: number of locules per pod; NPP: number of pods per plant; NSP: number of seeds per plant; StTh: main stem thickness (mm); SL: seed length; SWth: Seed width (mm); STh: seed thickness; TSW: total seed weight in the plant (g), W1000: 1000-seed weight (g), COEF J: evaluated in seed; coefficient J = (length./width, COEF H: evaluated in the seed, Coefficient H = (thick./width.); and YLD: yield in g/plot. (DOCX) [file pone.0188798.s004.docx]

**S4 Table.** Mean and standard deviation of the 23 agromorphological traits for the groups formed by the Ward method from the Mahalanobis distance for cultivars of the commercial group carioca.

| Traits^1/^ | Group I | | Group II | | Group III | | Group IV | |
| --- | --- | --- | --- | --- | --- | --- | --- | --- |
|  | Mean | σ | Mean | σ | Mean | σ | Mean | σ |
| PLL | 6.64 | 0.35 | 7.01 | 0.43 | 6.56 | 0.25 | 6.79 | 0.4 |
| PLW | 5.36 | 0.28 | 5.73 | 0.25 | 5.16 | 0.26 | 5.56 | 0.17 |
| PLI | 1.24 | 0.02 | 1.22 | 0.03 | 1.27 | 0.03 | 1.22 | 0.04 |
| CLL | 9.12 | 0.22 | 9.44 | 0.18 | 9.3 | 0.26 | 8.96 | 0.61 |
| CLW | 7.14 | 0.36 | 7.3 | 0.28 | 7.02 | 0.23 | 7.22 | 0.48 |
| CLI | 1.28 | 0.07 | 1.3 | 0.03 | 1.33 | 0.05 | 1.24 | 0.04 |
| StL | 84.6 | 8.1 | 98.48 | 3.56 | 82.44 | 6.81 | 58.75 | 14.53 |
| IFP | 15.34 | 0.95 | 18.06 | 0.15 | 14.98 | 0.69 | 14.64 | 0.9 |
| NN | 14.26 | 1.04 | 14.48 | 0.54 | 14.48 | 0.29 | 12.34 | 1.26 |
| PL | 11.29 | 0.19 | 11.67 | 0.78 | 11.55 | 0.16 | 10.61 | 0.43 |
| NSP | 6.2 | 0.28 | 6.03 | 0.55 | 6.03 | 0.16 | 5.56 | 0.09 |
| LP | 6.78 | 0.32 | 6.61 | 0.56 | 6.73 | 0.17 | 6.3 | 0.25 |
| NPP | 20.57 | 1.78 | 17.75 | 1.36 | 20.88 | 1.25 | 20.14 | 2.12 |
| NSP | 99.23 | 10.03 | 80.88 | 4.96 | 94.94 | 9.86 | 80.04 | 11 |
| StTh | 6.34 | 0.52 | 6.29 | 0.39 | 6.37 | 0.45 | 5.65 | 0.17 |
| SL | 10.52 | 0.32 | 11.22 | 0.24 | 10.75 | 0.15 | 10.73 | 0.17 |
| SWth | 6.94 | 0.1 | 7.1 | 0.25 | 6.67 | 0.06 | 6.74 | 0.08 |
| STh | 5.27 | 0.14 | 5.22 | 0.27 | 4.97 | 0.1 | 5.09 | 0.06 |
| TSW | 26.54 | 2.13 | 23.41 | 2.2 | 23.51 | 3.11 | 20.5 | 2.77 |
| W1000 | 268.34 | 7.7 | 292.33 | 22.23 | 247.99 | 10.32 | 255.84 | 7.51 |
| COEF J | 1.52 | 0.04 | 1.58 | 0.02 | 1.61 | 0.01 | 1.59 | 0.04 |
| COEF H | 0.76 | 0.02 | 0.74 | 0.03 | 0.74 | 0.02 | 0.76 | 0.01 |
| YLD | 2391.91 | 209.13 | 2265.08 | 288.84 | 2238.74 | 359.38 | 2215.06 | 250.68 |

^1/^PLL: primary leaf length (cm); PLW: primary leaf width (cm); PLI: primary leaf index (PLL/PLW); CLL: central leaflet length (cm); CLW: central leaflet width (cm); CLI: central leaf index (CLL/ CLW); StL: main stem length (cm); IFP: insertion height of the first pod (cm), NN: number of stem nodes, PL: pod length (cm); NSP: number of seeds per pod; LP: number of locules per pod; NPP: number of pods per plant; NSP: number of seeds per plant; StTh: main stem thickness (mm); SL: seed length; SWth: Seed width (mm); STh: seed thickness; TSW: total seed weight in the plant (g), W1000: 1000-seed weight (g), COEF J: evaluated in seed; coefficient J = (length./width, COEF H: evaluated in the seed, Coefficient H = (thick./width.); and YLD: yield in g/plot.
