# Supplementary material for: Distinctness of Brazilian common bean cultivars with carioca and black grain by means of morphoagronomic and molecular descriptors
Source: PLoS One. 2017 Nov 30;12(11):e0188798. doi: 10.1371/journal.pone.0188798 (PMC5708700; doi:10.1371/journal.pone.0188798)
Supplement: S5 Table — 1/PLL: primary leaf length (cm); PLW: primary leaf width (cm); PLI: primary leaf index (PLL/PLW); CLL: central leaflet length (cm); CLW: central leaflet width (cm); CLI: central leaf index (CLL/ CLW); StL: main stem length (cm); IFP: insertion height of the first pod (cm), NN: number of stem nodes, PL: pod length (cm); NSP: number of seeds per pod; LP: number of locules per pod; NPP: number of pods per plant; NSP: number of seeds per plant; StTh: main stem thickness (mm); SL: seed length; SWth: Seed width (mm); STh: seed thickness; TSW: total seed weight in the plant (g), W1000: 1000-seed weight (g), COEF J: evaluated in seed; coefficient J = (length./width, COEF H: evaluated in the seed, Coefficient H = (thick./width.); and YLD: yield in g/plot. (DOCX) [file pone.0188798.s005.docx]

**S5 Table.** Mean and standard deviation of the 23 agromorphological traits for the groups formed by the Ward method from the Mahalanobis distance for cultivars of the commercial group black.

| Traits^1/^ | Group I | | Group II | | Group III | | Group IV | |
| --- | --- | --- | --- | --- | --- | --- | --- | --- |
|  | Mean | σ | Mean | σ | Mean | σ | Mean | σ |
| PLL | 6.48 | 0.3 | 6.08 | 0.25 | 5.97 | 0.3 | 6.2 | 0.25 |
| PLW | 4.99 | 0.19 | 4.79 | 0.19 | 4.57 | 0.19 | 4.77 | 0.2 |
| PLI | 1.3 | 0.02 | 1.27 | 0.02 | 1.31 | 0.02 | 1.3 | 0.01 |
| CLL | 9.18 | 0.5 | 9.47 | 0.35 | 9.21 | 0.39 | 9.53 | 0.26 |
| CLW | 7.01 | 0.32 | 7.17 | 0.16 | 6.93 | 0.42 | 7.26 | 0.33 |
| CLI | 1.31 | 0.04 | 1.32 | 0.02 | 1.33 | 0.02 | 1.31 | 0.03 |
| StL | 77.73 | 4.31 | 85.04 | 6.27 | 90.04 | 3.87 | 89.67 | 2.31 |
| IFP | 15.07 | 1.25 | 15.54 | 0.97 | 17.71 | 1.43 | 17.72 | 1.14 |
| NN | 13.76 | 0.78 | 14.47 | 0.69 | 15.6 | 0.44 | 14.33 | 0.71 |
| PL | 10.22 | 0.57 | 10.4 | 0.27 | 9.1 | 0.1 | 11 | 0.7 |
| NSP | 5.93 | 0.31 | 6.49 | 0.18 | 6.25 | 0.03 | 6.19 | 0.15 |
| LP | 6.51 | 0.29 | 7.06 | 0.24 | 6.68 | 0.07 | 6.72 | 0.22 |
| NPP | 21.36 | 1.99 | 20.73 | 2.51 | 22.47 | 1.83 | 22.76 | 2.53 |
| NSP | 97.2 | 9.39 | 106.03 | 11.14 | 107.51 | 7.67 | 106.88 | 12.51 |
| StTh | 6.32 | 0.33 | 6.86 | 0.29 | 6.34 | 0.29 | 6.49 | 0.2 |
| SL | 10.59 | 0.22 | 10.34 | 0.19 | 9.64 | 0.13 | 10.31 | 0.35 |
| SWth | 6.76 | 0.09 | 6.63 | 0.04 | 6.33 | 0.1 | 6.23 | 0.09 |
| STh | 4.88 | 0.15 | 4.87 | 0.12 | 4.65 | 0.11 | 4.64 | 0.17 |
| TSW | 23.42 | 2.25 | 24.35 | 2.48 | 21.15 | 1.67 | 23.31 | 3.61 |
| W1000 | 239.69 | 15.28 | 227.29 | 7.18 | 195.06 | 0.1 | 216.85 | 17.65 |
| COEF J | 1.57 | 0.05 | 1.56 | 0.03 | 1.52 | 0 | 1.66 | 0.05 |
| COEF H | 0.72 | 0.03 | 0.73 | 0.02 | 0.74 | 0.01 | 0.74 | 0.03 |
| YLD | 1941.84 | 138.79 | 1748.5 | 182.75 | 1494.51 | 290.85 | 1757.52 | 94.68 |

^1/^PLL: primary leaf length (cm); PLW: primary leaf width (cm); PLI: primary leaf index (PLL/PLW); CLL: central leaflet length (cm); CLW: central leaflet width (cm); CLI: central leaf index (CLL/ CLW); StL: main stem length (cm); IFP: insertion height of the first pod (cm), NN: number of stem nodes, PL: pod length (cm); NSP: number of seeds per pod; LP: number of locules per pod; NPP: number of pods per plant; NSP: number of seeds per plant; StTh: main stem thickness (mm); SL: seed length; SWth: Seed width (mm); STh: seed thickness; TSW: total seed weight in the plant (g), W1000: 1000-seed weight (g), COEF J: evaluated in seed; coefficient J = (length./width, COEF H: evaluated in the seed, Coefficient H = (thick./width.); and YLD: yield in g/plot.
